# Large-scale and cost-efficient agrivoltaics system by spectral separation

**DOI:** 10.1016/j.isci.2023.108129

**Published:** 2023-10-05

**Authors:** Fangxin Zhang, Ming Li, Wei Zhang, Wenjun Liu, Altyeb Ali Abaker Omer, Zhisen Zhang, Jianan Zheng, Wen Liu, Xinyu Zhang

**Affiliations:** 1 Department of Optics and Optical Engineering, School of Physical Sciences, University of Science and Technology of China, 96 Jinzhai Road, Hefei City 230026, China; 2 Xiong’an Institute of Innovation, Xiong’an New Area 071700, China

**Keywords:** Physics, Optics, Agricultural engineering

## Abstract

Agrivoltaics (AV) offers a promising solution to address both food and energy crises. However, crop growth under photovoltaic (PV) conditions faces substantial challenges due to insufficient light transmission. We propose a large-scale and cost-effective spectral separated concentrated agricultural photovoltaic (SCAPV) system. The system utilizes concentrator modules, cell components, and dual-axis tracking systems to enhance power conversion efficiency (PCE), achieving a maximum PCE of 11.6%. After three years of successful operation, a 10 kWp power plant achieved an average annual electricity generation exceeding 107 MWh/ha. The results showed higher yields of various crops, including ginger and sweet potatoes, and significant improvements in soil moisture retention compared to open air. The improvements in PCE and microclimate validate the scalability of the SCAPV, which provides better plant conditions and cost-effectiveness, with an estimated cost reduction of 18.8% compared to conventional PV power plant. This study provides valuable insights and directions for improvement in AV.

## Highlights

• A 10 kWp SCAPV successfully runs for 3 years with normalized 107 MWh/ha power
• The SCAPV system improves maximum power conversion efficiency up to 11.6%
• The benefits of SCAPV system for several crops yield and microclimates are proved
• This study implies that SCAPV technology is cost-effective

## Graphical abstract

**Figure:**
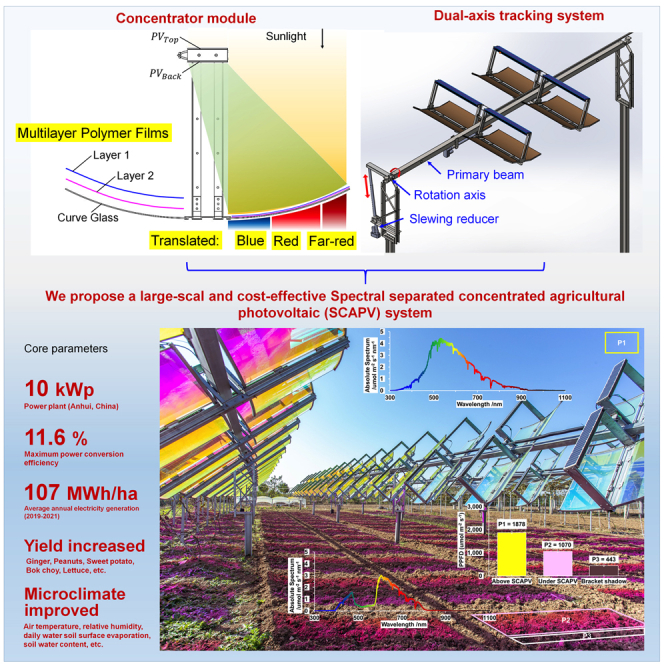

## Introduction

The concept of agricultural photovoltaic (APV) systems, which is also known as agrivoltaics (AV), originated from the idea of coexistence of power generation and crop cultivation by Goetzberger and Zastrow in 1982.^1^ Since 2017, AV has been recognized as a successful strategy for avoiding or mitigating land impacts from photovoltaic (PV) systems in the Global Land Outlook, which focuses on energy and land use by International Renewable Energy Agency (IRENA) and United Nations Convention to Combat Desertification (UNCCD).^2^^,^^3^^,^^4^

**Figure 1:**
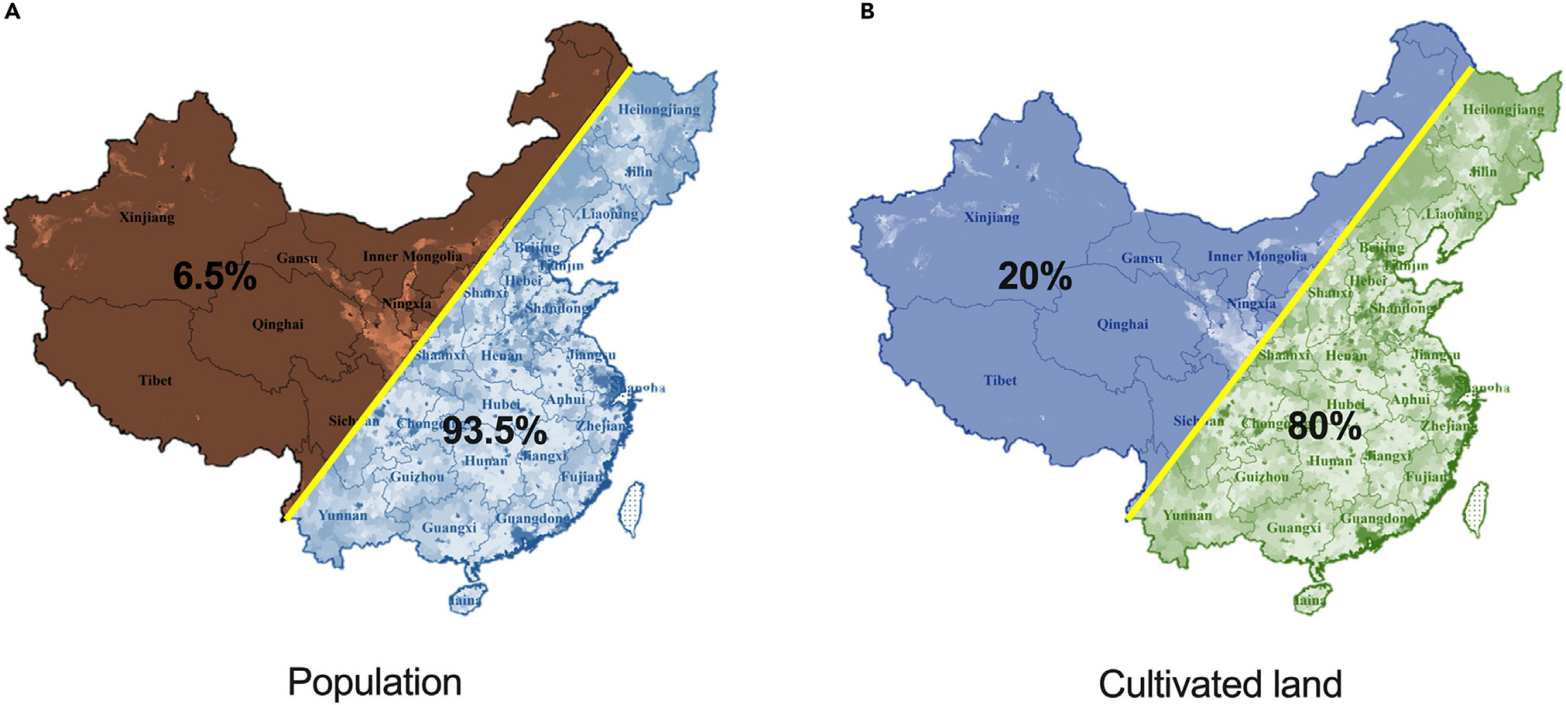
The proportion of population and cultivated land in southeast and northwest China bounded by the Hu Line (A) The population. (B) The cultivated land.

Capitalizing on technology and yield effects, China has experienced a 90% reduction in the cost of PV panels over the past decade. Compared to the thermal power cost of 0.27 Renminbi (RMB)/kWh,^5^ the cost of a PV plant located in the Gobi Desert of Northwest China has been reduced to as low as 0.13 RMB/kWh.^6^ Figure 1A shows the concentration of the populace in the southeast, an estimated 93.5%, if Hu Line^7^ is the boundary. Using ultrahigh voltage transmission, PV-generated electricity from the northwest would be transmitted to heavily populated, industrialized southeastern regions, causing an increase in cost per kWh by more than double, for which demand of a locally consumable distributed energy supply was emphasized.

As shown in Figure 1B, the majority of China’s cultivable land is located southeast of the Hu line. AV in China benefits from the high demand for electricity and boasts a national average grid-connected price of 0.39 RMB/kWh, varying from 0.35 to 0.45 RMB/kWh across provinces. With the addition of government subsidies, the gross domestic product (GDP) can increase up to 50,000 RMB/mu, 10 times more than that of traditional farming. From 2015 to 2020, the Chinese government utilized the AV station for anti-poverty initiatives, resulting in the construction of over 80,000 village-level PV power plants with a combined capacity of more than 28.6 GWp. These power plants were mainly located in the central-eastern region and aided over 10 million rural people who were living in poverty.

The increased installed capacity of PV necessitates vast land resources; thus, the development of AV on limited lands needs to coexist in harmony with both ecology and crops, particularly in ecologically fragile areas. The key challenge for AV is the insufficient light underneath PV modules, as the intensity in the shadows is only 32%–52% of that of solar energy.^8^^,^^9^^,^^10^ Additionally, it is generally lower than the needed photosynthetic photon flux density (PPFD) for most crop growth. Mosaic PV or strip transparent PV modules have been put forward and implemented in AV^11^ to ensure the growth of crops' PPFD. However, the cost of these types of AV is appreciably higher since they reduce the power generation per unit area.

Spectrally separated APV (SAPV) has emerged as a promising solution for enhancing energy utilization efficiency when compared to mosaic PV, which is a geometric spectroscopic solution. As early as 1955, Jackson ED, an American scientist, proposed the idea of separating different wavelengths of light and irradiating them onto different PV cells.^12^ In 1978, American scientists Moon et al. realized this idea by using III-V cells in practice.^13^ With the emergence of PV cells of various materials and spectral filters, several researchers have studied SAPV systems.^14^^,^^15^^,^^16^^,^^17^^,^^18^^,^^19^^,^^20^^,^^21^^,^^22^ However, these studies had focused on small-scale greenhouses and laboratory modules. The efficiency of these systems is notably lower than that of ordinary crystalline PVs, and there have been no systematic successful reports on large-scale construction, spectral microclimate, or spectral agricultural planting results. Table 1 summarizes recent years on SAPV.

**Table 1:** SAPV reported in the literature

State	Materials for spectral separation	Wavelength of spectral separation	PV cell	PV efficiency
Netherlands^14^	Metallic multilayered material (SOL-MOX Hilite Ò from Bekaert, Zwevegem, Belgium) and a dielectric multilayered based on plastic film (Ebiral from 3M, St. Paul, USA)	Transmit PAR and reflect NIR	Crystalline silicon (C-Si)	3.3%^a^
United States^15^	Polymethyl methacrylate (PMMA) plastic embedded with luminescent perylene red dye	Absorb some of the blue and green wavelengths of the solar spectrum but transmit the remaining wavelengths	C-Si narrow PV strips	4.0%^a^
China^11^	Multilayer dichroitic polymer film	Transmit blue light between 397 nm and 492 nm and red light between 604 nm and 852 nm	C-Si concentration PV	6.8%^a^
Israel^16^	Flexible organic photovoltaic material	Approximately 20% transmissivity, 15% reflectivity, and 65% absorptance in the photosynthetically active radiation (PAR) range	Organic solar cells	0.8%^a^
China^17^	Wavelength selective semitransparent polymer	A weak absorption for blue and red light but an intense absorption for green and NIR light	Semitransparent polymer solar cells (ST-PSCs)	7.75%^b^
United States^18^^,^^19^	Semitransparent organic solar cells	The OSC provides an opportunity to manage light transmittance from the UV to the infrared (IR).	Semitransparent organic solar cells (OSCs)	4.9%^b^
United States^20^	Array of micro-crystalline silicon nanowires	Absorbs almost all green light but absorbs almost no blue and red light	Spectrally selective solar cells consist of an array of inorganic optical antennas	4.2–7%^c^
United Kingdom^21^	Tinted semitransparent solar panels	Absorb preferentially blue and green light, leaving most of the red for photosynthesis	Dye-Sensitized solar Cells (DSCs)	8%^b^
China^22^	SAVE AU60 CH thin film produced by American RAYNO C pany is used as spectral splitting film (SSF)	Transmits visible light and converts near-infrared light into electricity	Triple-junction solar cells (GaInP/GaAs/Ge)	6.88%^a^

a PCE was determined by measuring the I-V characteristics and solar radiation through a source meter and light intensity meter in the open sunlight environment.

b PCE was determined by measuring the I-V characteristics from source flow meters and solar simulator in the laboratory.

c PCE is derived from theoretical calculations, not from experimental tests.

We introduced the SCAPV model in 2018. The model uses the principle of interference filtering to achieve spectral separation of sunlight based on the difference in spectral response of PVs and photosynthesis. The red and blue wavelengths are transmitted for photosynthesis as they match the absorption peaks of plant chlorophyll, while all other wavelengths are reflected for concentrated power generation. SCAPV has the potential to address both power generation and land use issues. An experimental installation consisting of three concentrator modules constructed in 2018 produced the highest system efficiency of 8.8%.^11^ It was verified under a small-scale (approximately 3 m^2^) experimental film that crops, such as lettuce, did not experience a decrease in yield when grown under the system.^23^

In this study, we have significantly improved the maximum power conversion efficiency (PCE) of SCAPV, reaching a remarkable 11.6%. Our approach involved utilizing the modular design of concentrator module, optimizing the cell components and the concentrating curve, and enhancing the dual-axis tracking system (DATS). Furthermore, we constructed a pioneering 10 kWp SCAPV farm, the largest-scale and commercially operated SAPV system to date, which has remained stable for over three years. In addition, we conducted extensive experimental investigations of crop growth and microclimate on a large-scale experimental farmland with a spectral separation environment. This research has critical implications for diverse forms of SAPV, and not exclusively for SCAPV.

Having constructed such an SCAPV farm, we found that the system cost could be reduced below that of conventional PV systems. In this paper, we dedicated a section to analyzing the costs of the tracking system, the curved glass panel, and the film and compared them to conventional PV systems. These comparisons may accelerate the adoption of SAPV technology in the future.

### System design and methods

#### Design of mass-produced concentrator module for SCAPV

This paper presents the core components of the SCAPV system, which include concentrator module, DATS, and bracket. The concentrator module is the key device for spectral separation, concentration, and power generation. It comprises a concentrating curved glass (CCG), a multilayer polymer film (MPF), a high-efficiency concentrator PV cell capable of withstanding the light intensity of the concentrator, and an efficient heat dissipation structure. A total of 128 concentrator modules were manufactured using a modular design.

According to international standards, concentrated photovoltaics (CPVs) are classified into high concentrations with concentration ratio (CR) ≥ 100, medium concentrations (5 ≤ CR < 100), and low concentrations (CR < 5). For low concentrations, light is typically concentrated using C-Si cells, whereas medium concentrations mainly use single-junction or double-junction III-V cells. High concentrations, on the other hand, usually employ triple-junction and quadruple-junction III-V cells. InGaAsP quad-junction cells, with the capacity to tolerate up to 1,000 times concentrated light, are already available. These cells, which have efficiency ratings that reach 40% and are sized up to 4.5 × 4.5 mm^2^, have been developed.^24^^,^^25^

To meet economic feasibility and PCE criteria while reducing the need for accurate tracking and heat dissipation, we utilize new C-Si cells at low CR. Under constant temperature conditions, studies have demonstrated that C-Si cells are able to maintain a linear correlation between output power and CR at CR below 6.^26^ The CCG used as a concentrator reflector in this study is ultra-white and toughened. The selection process took into account the spectral shift characteristics of the MPF, processing cost, module size, holder height, and cell compatibility, ultimately resulting in the choice of a medium concentration solution (CR = 6).

**Figure 2:**
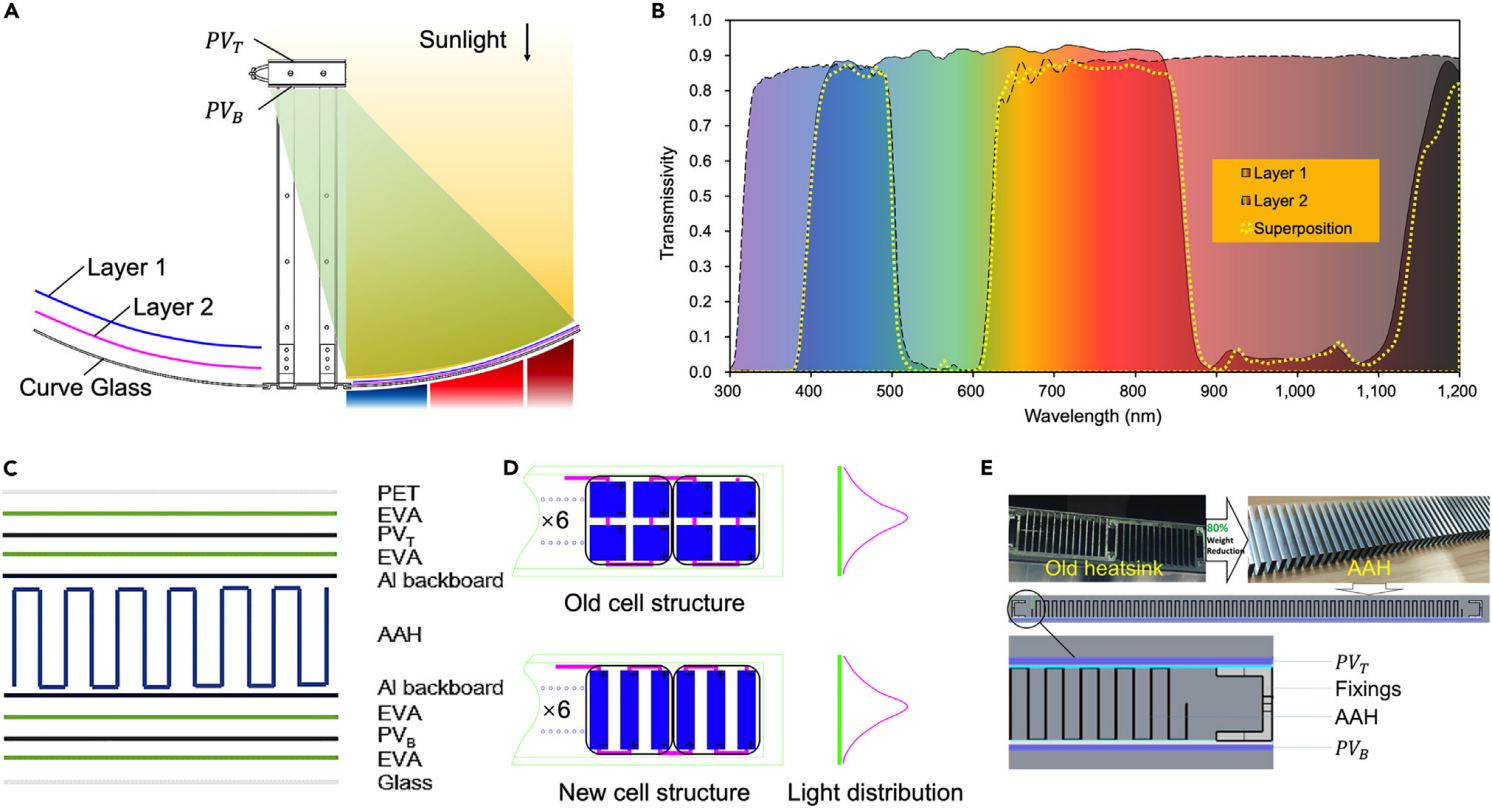
Spectral separation principle and structure of the concentrator module (A) Principle of the optical path of the concentrator module with two layers of MPF attached. (B) Transmission spectrum of layer 1, layer 2, and superposition (3700DUV spectrophotometer, Shimadzu, Japan).4. (C) A schematic diagram of the laminated structure of the PV module. (D) Series structure of PV cells with different cutting sizes and light intensity distribution on their surfaces. (E) Schematic diagram of the structure of the modified heat sink and support frame.

Supplementary Material S1 outlines the design process for the reflector of the concentrator module. We constructed the final reflector using the curve from Figure S1D, with the MPF attached, as depicted in Figure 2A. Spectral separation relies on the MPF, the most crucial material, which achieves light filtering by using the interference principle on the surface of high- and low-refractive-index materials. Despite not reaching mass production, we employed layer 1 (Dazzle Film, 3M, USA) and layer 2 (PR-90, 3M, USA) to achieve transmissions for the blue light band (397 nm–492 nm) and the red-far red light band (604 nm–852 nm) in this project, as depicted in Figure 2B. We established our production line for these films for pilot testing.^27^ Figure 2B illustrates the transmission spectrum.

SunPower Maxeon Gen II PV cells (125 mm × 125 mm) were selected for the PV module. These cells were cut into 1/3 strip PV cells (41 mm × 125 mm) and then connected in series using ethylene vinyl acetate (EVA) along with PV glass and a backboard, resulting in the formation of PV modules consisting of 24 such strip PV cells. Figure 2C depicts the lamination structure of the PV module. The PV module consists of two parts: the backside PV (${PV}_{B}$), which receives concentrated sunlight from the concave screen, and the top PV (${PV}_{T}$), which receives direct sunlight. We previously used square concentrator cells (41 mm × 41 mm) and encountered issues with inconsistent sunlight intensity caused by minor variations in tracking position, which ultimately led to non-uniform current output among the solar cells and affected overall current output as shown in Figure 2D. Therefore, we adopted 1/3 strip PV cells (41 mm × 125 mm) to mitigate the requirements for high tracking accuracy, as this type of solar cell exhibits relatively minor changes in sunlight intensity captured by each individual cell in response to variations in tracking position, thereby improving the consistency of current output. The rationale behind the selection of the type, structure, and experimental results is discussed in results section. The system also utilizes an integrated bent aluminum alloy heat sink (AAH) shown in Figure 2E, which is 80% lighter than the traditional cast heat sink.

#### Design of DATS

**Figure 3:**
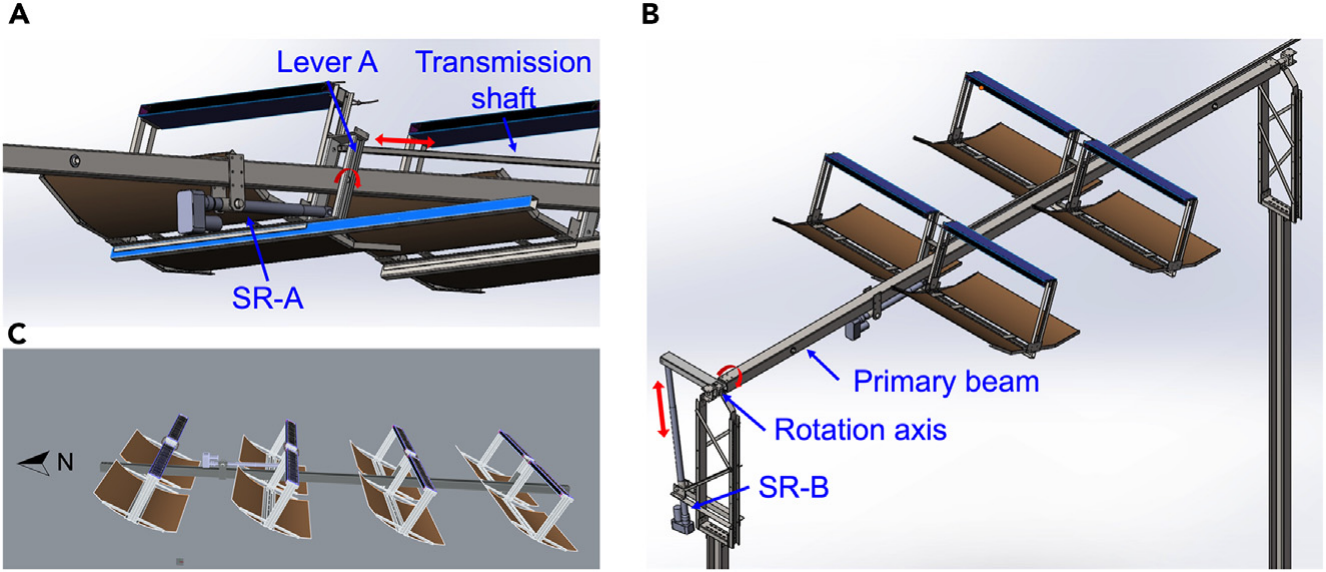
Simulation of the installation of concentrator modules into a DATS (A) SR-A drives the Lever A to achieve solar altitude tracking for multiple concentrator modules. (B) SR-B drives the primary tracking axis to achieve solar azimuth tracking for multiple concentrator modules. (C) Multiple concentrator modules are fixed in the primary tracking axis and arranged in a north-south direction.

The project utilized SolidWorks software to design a DATS, which was achieved by constructing a mechanism model, as shown in Figure 3. To achieve dual-axis tracking, the system relies on two motors to control the angle of the spotting module for both azimuth and altitude angle tracking, utilizing only one support axis.

In Figure 3A, a one-dimensional servo system is depicted with slewing reducer A (SR-A) rotating two groups of concentrator modules for solar altitude angle tracking through Lever A. The concentrator modules on the entire main beam square tube are driven via a drive shaft. Integral brackets on the lower part of the concentrator module support the weight of the concentrator modules and wind/snow loads, and the weight is transferred to the primary beam through the north-south swing arm rotation shaft and the swing arm weldment. Figure 3B illustrates the slewing reducer B (SR-B) controlling solar azimuth tracking by rotating the primary beam east-west through a rotation shaft. A programmable logic controller (PLC) circuit controls both SR-A and SR-B to achieve solar altitude angle and azimuth tracking using astronomical algorithms.^28^

**Figure 4:**
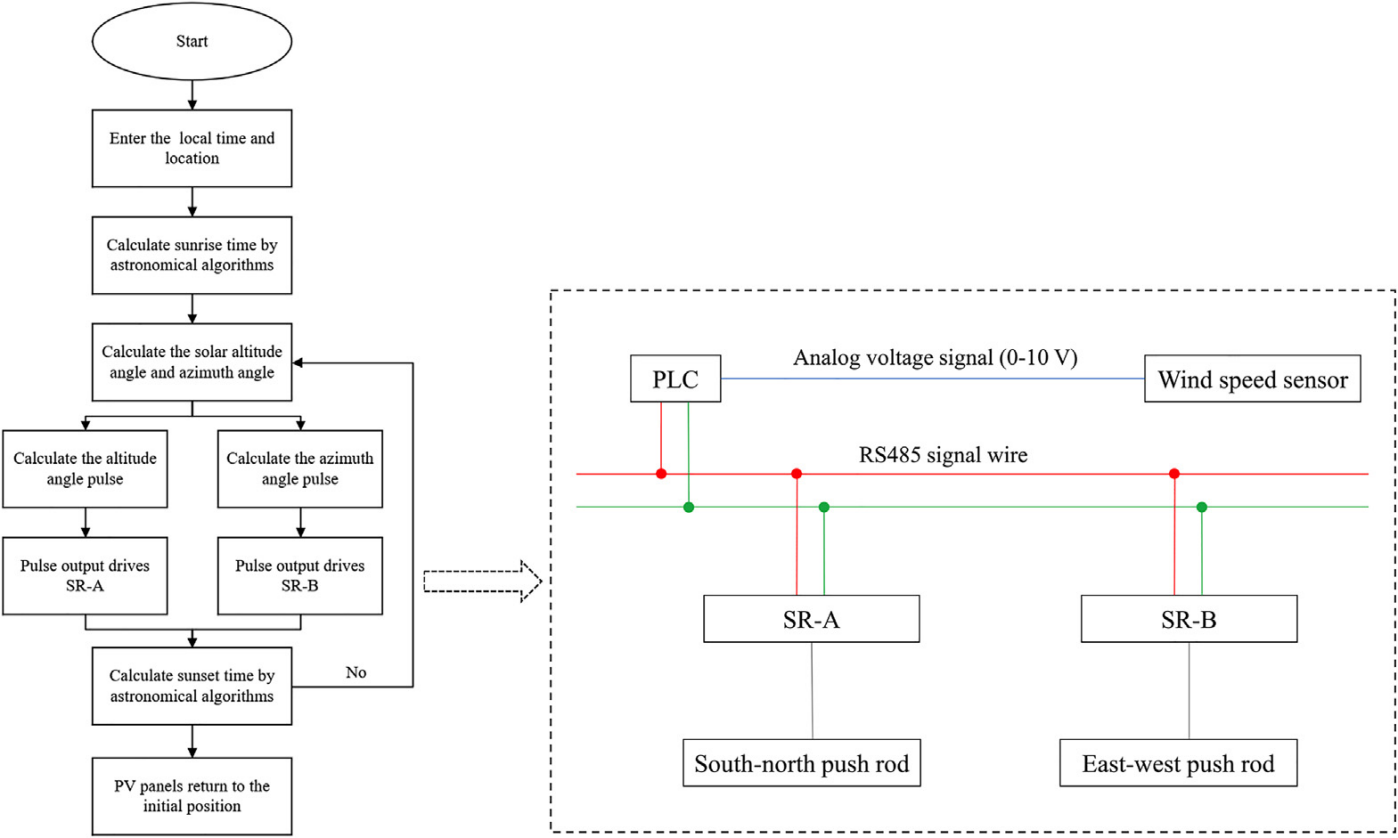
Tracking system control flow chart

The DATS algorithm consists of several steps as follows: (1) calculation of the solar altitude and azimuth angle for each day based on the location and time zone of the APV system; (2) generation of a tracking data table containing solar altitude, azimuth angle, date, and time based on the calculated values; (3) control of the system by the PLC based on the local time and location using a flow chart presented in Figure 4. (4) adjustment of the optimal position of the east-west and north-south axes of multiple concentrator modules.

#### Construction of the large-scale SCAPV system

The above structure was utilized by this paper to construct a sizable SCAPV station. Figure 3C shows the primary beam aligned in a north-south direction with 32 concentrator modules arranged in 16 groups at 1.5 m intervals for a balanced structure. SR-A controlled the solar altitude angle (±45°) of all 16 groups in a link, and SR-B allowed the primary beam to steer overall solar tracking for azimuth direction (0°–60°). The height of the primary beam from the ground is 2.5 m, and each concentrator module has an equivalent installed power of 80 Wp provided by ${PV}_{B}$ and ${PV}_{T}$, thus yielding 2.56 kWp total installed power in a row.

**Figure 5:**
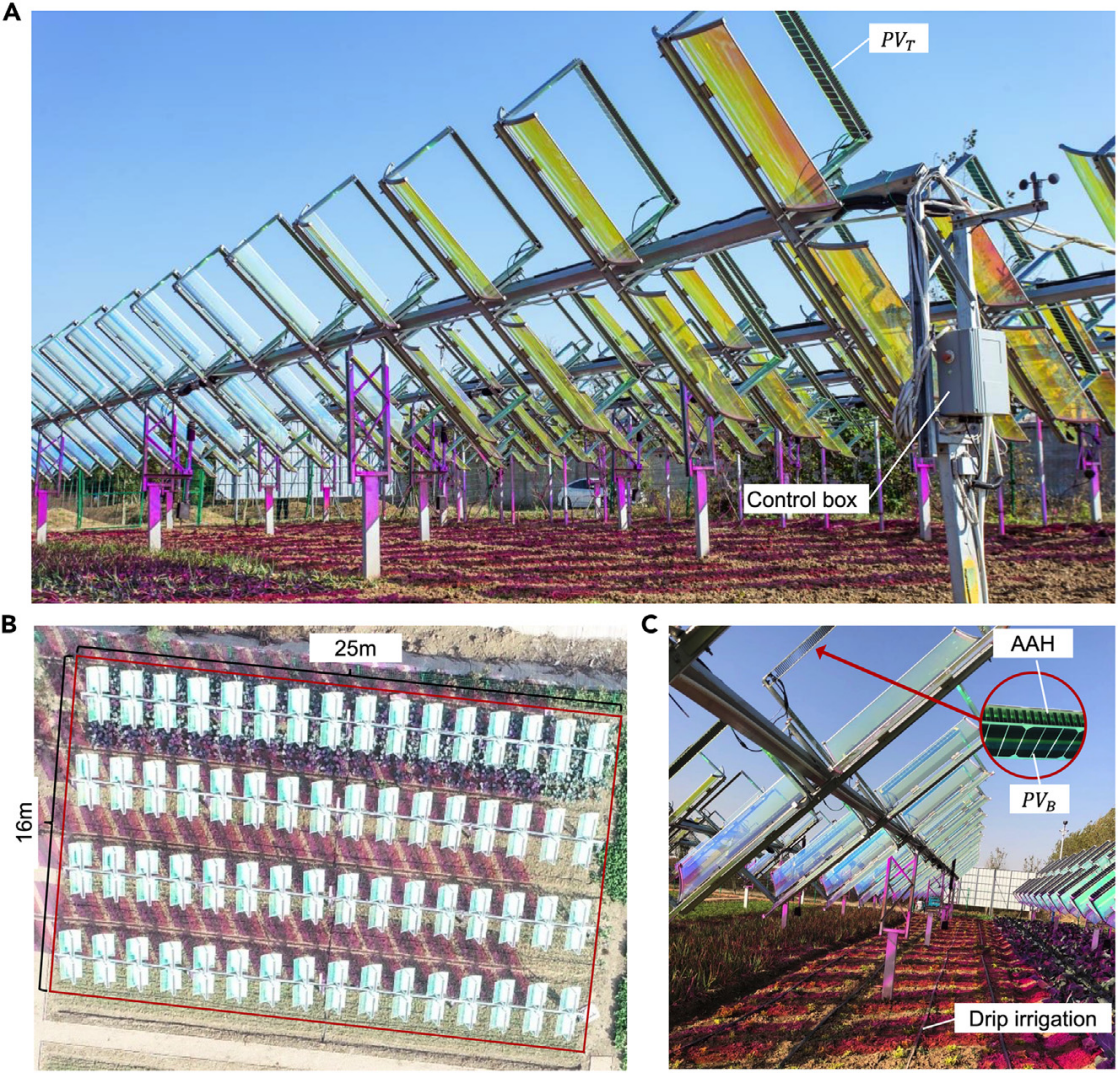
The completed 10 kWp SCAPV system (Fuyang city, Anhui, China) (A) One row of SCAPV system. (B) Top view. (C) Side view.

In 2019, we constructed a sizable SCAPV system by arranging four columns of the rows with 4 m intervals. As shown in Figure 5, the system has a total installed capacity of 10.24 kWp and covers an area of 400 m^2^, making it the first large-scale and maximal SAPV application base reported in the literature to date.

## Results

### Performance of the sizable SCAPV system

This study details the establishment of a 400 m^2^ SCAPV power generation system in Fuyang, located in the Anhui Province, China (115.9 E, 32.9 N). The system was planned and erected in early 2019, connected to the alternating current (AC) inverters, and subsequently tested for both power generation and crop cultivation experiments from 2019 to 2021. For over 1,200 days, the system was consistently grid-connected, accumulating 10 MWh in power generation.

**Figure 6:**
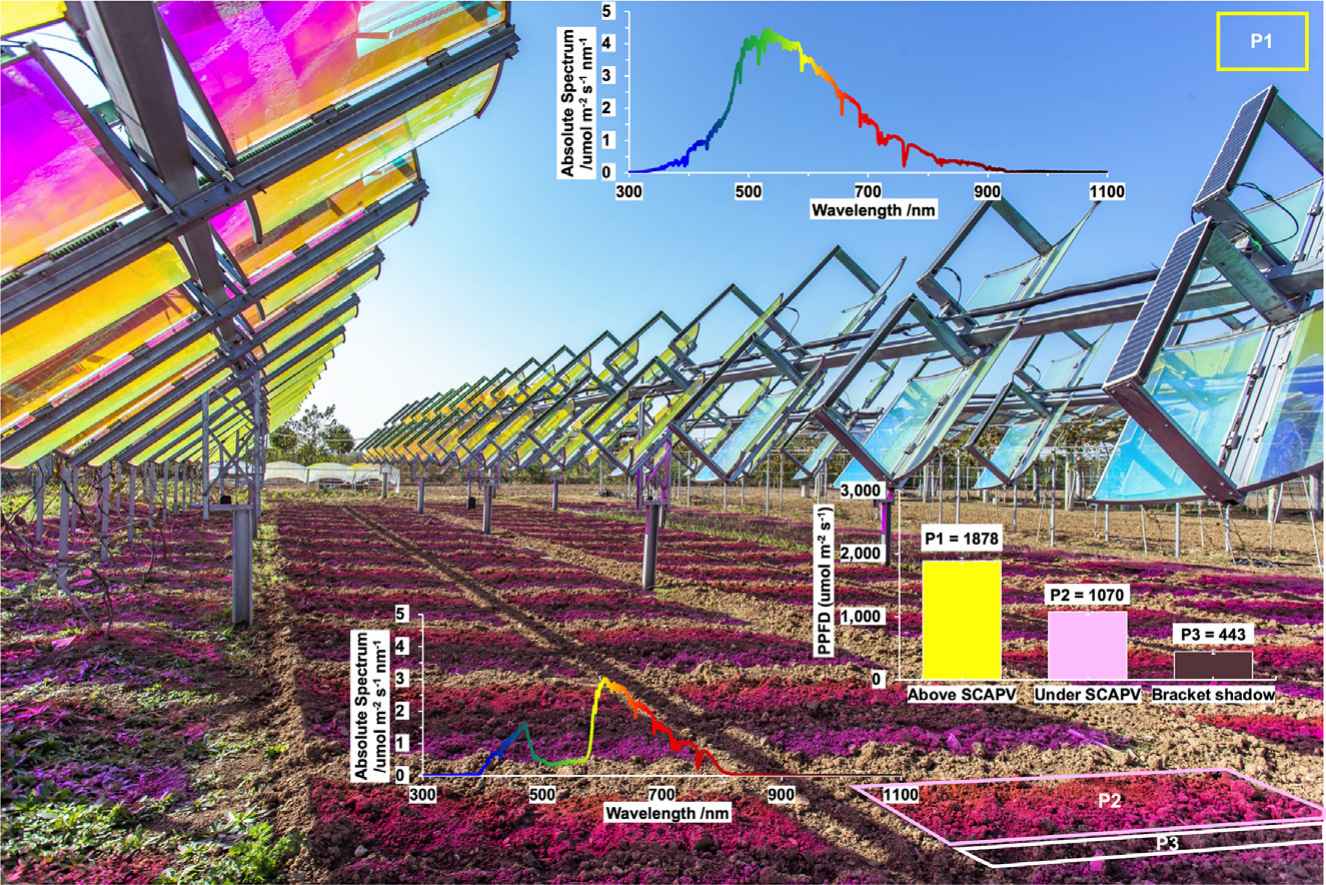
Spectrum and light intensity of the bottom of the SCAPV system compared to the open air

The height of the SCAPV system is set at 2.5 m from the ground in a horizontal position, making it compatible with small farm machinery. The MPF is used to allow the sunlight that meets the requirements for plant growth to pass through to the SCAPV. The sunlight above the SCAPV is defined as P1, while the sunlight below the MPF of the SCAPV is defined as P2. The light intensity below the non-transparent parts of the PV bracket or cells is defined as P3. Specifically, on July 22, 2019, the measured light intensity under SCAPV (P2) was 57% of that measured in the open air (P1), and 24% of that measured in the shade under the PV modules (P3), as illustrated in Figure 6. Moreover, from the spectrum at P1 and P2, observing a purplish-red hue on the ground attributed to the transmission of the red and blue light bands. This phenomenon is commonly observed in plant factories that use artificial lighting and aligns with the absorption spectrum of plant photosynthetic pigments, including chlorophyll *a* and chlorophyll *b*.

### PV cell selection and efficiency

Under specific sunlight intensity, the electrical parameters of the electricity generated by the PV system are determined by the PV cells, including the open circuit voltage (OCV), short circuit current (ISC), and maximum power point (MPP). Currently, GaAs multi-junction cells have garnered attention for their ultrahigh efficiency compared to C-Si cells, and low PV usage in concentrating power systems makes them an ideal cell for this specific application, despite their high cost. In this study, the practicality of GaAs cells and interdigitated back contact silicon (IBC-Si) cells (SunPower, USA) were tested under the same irradiance conditions specified by the normalized standard test conditions (STCs), wherein an equivalent cell efficiency per unit area power meter was derived. Results showed that GaAs cells exhibited an excellent power generation efficiency of 35%, whereas IBC-Si cells were only 23% efficient, as presented in Table 2.

**Table 2:** Comparison of efficiency between GaAs and IBC-Si cells under normalized STC

Cells	Solar irradiance (w/m^2^)	OCV (V)	ISC (A)	MPP (W)	Vmax (V)	Imax (A)	Power per unit area	Equivalent cell efficiency
GaAs	872.0	7.6	0.3	2.2	6.7	0.3	350.4	35%
IBC-Si	868.0	2.0	4.2	3.1	1.6	1.9	226.0	23%

**Figure 7:**
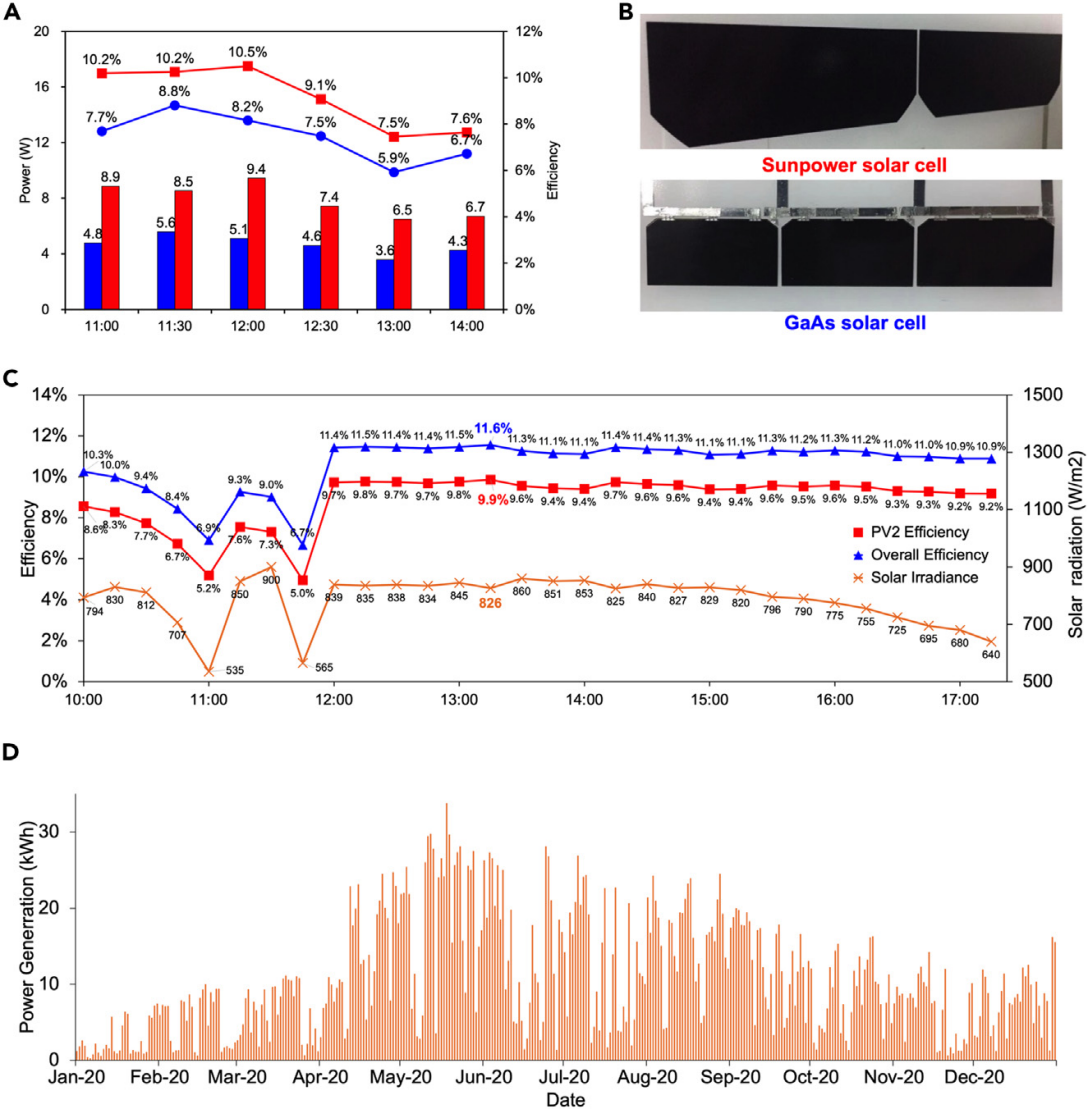
SCAPV system power parameters (A) Efficiency measured using GaAs and SunPower cells. (B) GaAs cells and IBC-Si cells. (C) The maximum PCE of the SCAPV system increased to 11.6%. (D) System average daily power generation in 2021.

Subsequently, efficiency tests were conducted on both the GaAs and IBC-Si cells integrated into concentrator module located at ${PV}_{B}$. The results of these tests are presented in Figure 7A. Due to the high transmission rates of red and blue light, with the remaining light being reflected and concentrated, the efficiency of the GaAs cell decreased noticeably, with an average efficiency of only 8.8%. Conversely, the IBC-Si cell achieved an efficiency of 10.5%, demonstrating superior concentrated power generation capability.

### Efficiency of concentrator module

APV systems designed using spectral separation in recent years have been less efficient than expected, with no reported cases of efficiency surpassing 10%, as demonstrated in Table 1. In 2018, we measured a PCE of approximately 8.8% for the small-scale SCAPV prototype we developed, as indicated in prior research.^11^

The overhead efficiency of the concentrator module is subject to fluctuation due to changes in the proportion of directly scattered light. The system efficiency comprises two components: the efficiency of ${PV}_{B}$, denoted as ${PCE}_{B}$ and the efficiency of ${PV}_{T}$, denoted as ${PCE}_{T}$. The sum of the two components constitutes the PCE. On July 22, 2019, we conducted measurements of the total solar radiation, ${PCE}_{B}$, and PCE every 30 min and recorded the data in Figure 7C. Notably, $PCE_{B}$ was low and highly variable during the morning due to cloudy weather. However, under clear afternoon weather, a maximum ${PCE}_{B}$ of 9.9% and maximum PCE of 11.6% were recorded through the day, both of which improved substantially from the 2018 test results and upper levels reported in the literature regarding the same field of SAPV technology, as presented in Table 1.

### Power generation of the SCAPV system

From 2020 to 2021, the SCAPV system operated without issue and generated a total of 7,787 kWh of electricity through inverter maximum power point tracking (MPPT) statistics: 3,872 kWh in 2020 and 3,915 kWh in 2021, with unit power annual generation capacities of 0.377 kWh/year and 0.382 kWh/year, respectively. Currently, under full sunlight exposure, regular C-Si cells generate a mere 1.1 kWh/year. Notably, based on the SCAPV system’s actual light intensity measurement data, approximately 43% of the total solar radiation contributes to power generation, corresponding to a radiation-converted unit power annual power generation capacity of 0.473 kWh/year for ordinary C-Si cells. Thus, SCAPV efficiency has reached 80% of that for regular C-Si cells.

Figure 7D illustrates the daily power generation of the system in 2020 over four seasons corresponding to 36%, 34%, 18%, and 12% of the year, with the highest power generation of 33.8 kWh/day occurring on May 12.

### Crop planting results

We have previously conducted experiments on three vegetables (lettuce, cucumber, and water spinach) under MPF in the laboratory. The results indicated that the leaves of the three vegetables had superior soluble sugar and photosynthetic rate indicators, and the overall plants were taller and heavier.^23^ However, the effect of large-scale open-air cultivation has not yet been verified. In this study, we conducted cultivation of different crops in an outdoor spectrally separated environment to verify the optical feasibility and generalizability of the solar spectral separation technology for crop cultivation. The results demonstrated higher yields for ginger, peanut, sweet potato, bok choy, and lettuce. The yield increase rates ranged from 6.5% to 47.9% under SCAPV compared to open-air cultivation, as shown in Table 3. Overheating in summer led to lower yields in open-air cultivation of lettuce, while growth under SCAPV was normal. The overall results were positive, and although non-photosynthetic light reflected to generate electricity resulted in lower radiant power under SCAPV, preliminary indications are that crop yields did not decrease, but increased. Therefore, this may have little effect on the plant in terms of photosynthesis. However, the microclimate under which the plant is exposed exhibited noticeable changes relative to open-air cultivation, and this may be the main reason for the change in crop yield under SCAPV. The microclimate data that follow can provide an indication. We have mainly shown the yield results of different crops after spectral separation. However, in terms of statistically analyzing experiments in agricultural cultivation, we realize that further research and data statistics are needed. In order to explore issues related to agricultural experiments in more depth, our next step will be to conduct more comprehensive and detailed agricultural experiments with statistical analyses.

**Table 3:** Yields of large-scale crops grown under the SCAPV system（1 mu = 667㎡）

Crop	Open air (CK)			SCAPV			Proportion
	Area (m^2^)	Yield (kg)	Equivalent yield per mu (kg)	Area (m^2^)	Yield (kg)	Equivalent yield per mu (kg)	
Ginger	100	294.0	1960.8	100	319.0	**2127.5**	**108.5%**
Peanuts	15	6.1	271.1	15	6.5	**288.9**	**106.5%**
Sweet potato	100	322.9	2153.8	70	294.2	**2522.0**	**117.1%**
Bok choy	8	29.2	2433.5	8	32.3	**2691.8**	**110.6%**
Lettuce	7	16.3	1552.5	7	24.1	**2295.4**	**147.8%**

### Microclimate and soil moisture capacity under SCAPV

**Figure 8:**
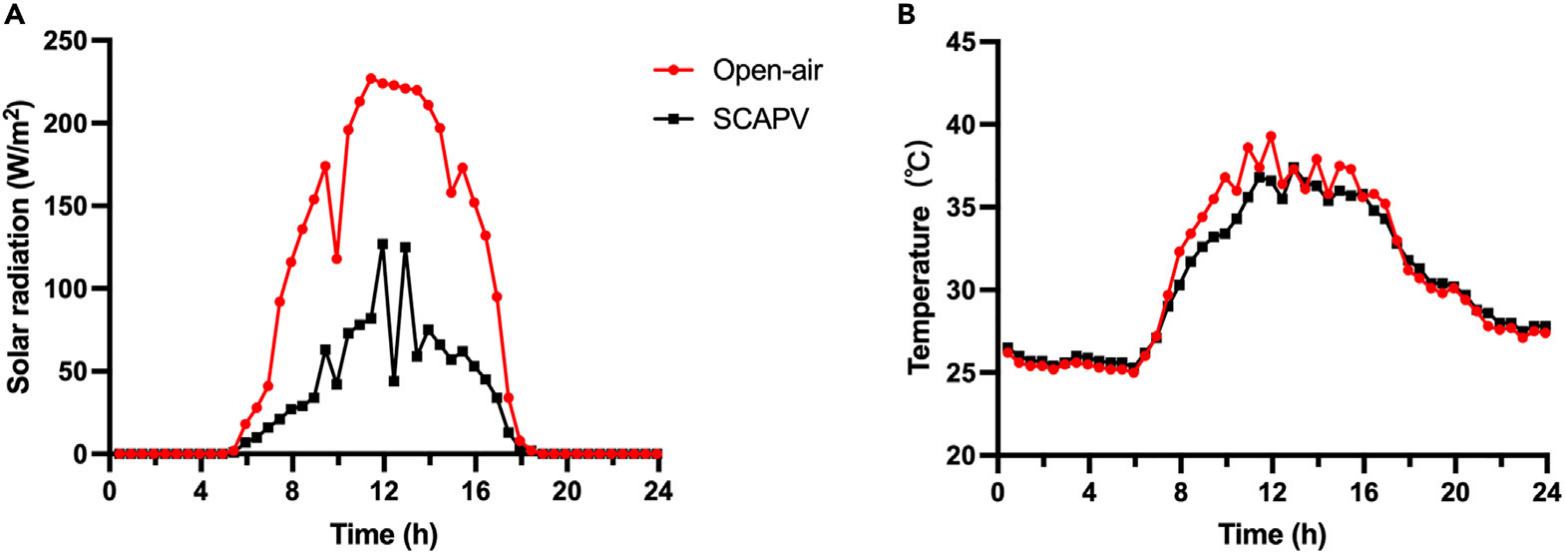
Hourly solar radiation and temperature in the open air and under the effect of SCAPV treatment (A) Solar radiation. (B) Temperature.

In this study, by measuring light and temperature variations at half-hourly intervals on a whole day, it was observed that light radiation under SCAPV was noticeably lower during the day as shown in Figure 8A. At the same time, daytime temperatures under SCAPV were relatively low, especially in the morning, and comparable or slightly lower in the afternoon as shown in Figure 8B. The slightly lower temperatures under SCAPV are supposed to favor water evaporation and photosynthetic efficiency.

**Figure 9:**
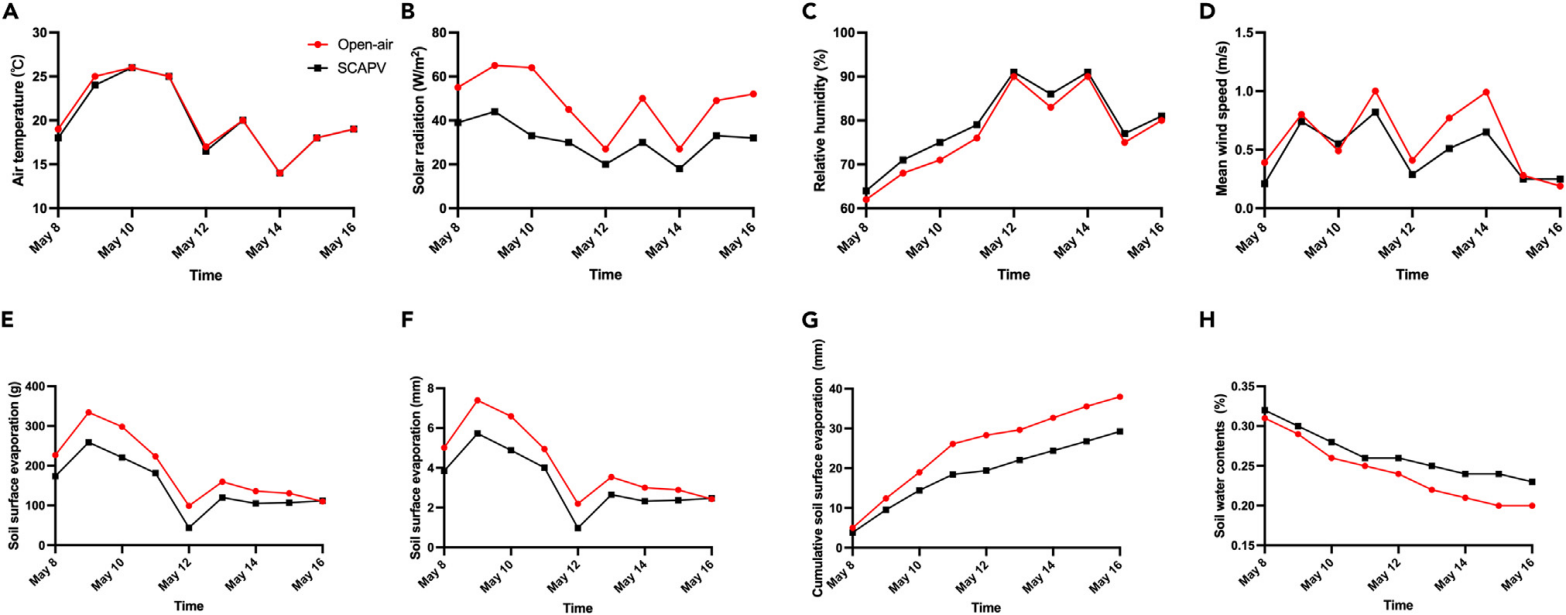
Weather parameters and water surfaces evaporation collected using data loggers under the effect of open-air and SCAPV treatments (A) air temperature (ºC). (B) solar radiation (W/m2). (C) relative humidity (%). (D) wind speed (m/s). (E) daily water soil surface evaporation (g). (F) daily water soil surface evaporation (mm). (G) cumulative water soil surface evaporation (mm). (H) soil water content (%).

The evaporation rate of the soil surface is affected by soil water content and weather conditions like solar radiation, wind speed, air temperature, and humidity.^29^ This experiment exposed the weather daily average conditions directly to the soil surface of control treatment (CK) and SCAPV, as shown in Figures 9A–9D. The air temperature during the experiment period in two treatments is shown in Figure 9A, indicating the relationship between air temperature and solar radiation. When the temperature increases, the solar radiation also increases, as illustrated in Figures 9A and 9B. As shown in Figure 9C, water evaporation is high under CK when the solar radiation is high, while it is low under SCAPV. Therefore, polymer multilayer film (PMF) allows only spectrum wavelengths necessary for plant photosynthesis while blocking the other unnecessary wavelengths.

Figures 9E–9H illustrate the cumulative soil surface evaporation process over time for both treatments. Cumulative soil surface evaporation over nine days was 37.99 mm in the CK and 29.25 mm under SCAPV. The partial sunlight transmitted by the PMF noticeably reduced cumulative evaporation. Throughout the nine days, the CK had the highest cumulative evaporation compared to SCAPV, resulting in a 23% reduction in water evaporation by SCAPV compared to CK.

Due to the partial sunlight, soil water evaporation was limited during the experiment period under SCAPV, allowing soil surface content to be optimized. Figure 9H illustrate the gradual loss of soil water content, which by the end of the nine days was conserved by 9%, compared to that of CK. The findings support that the influence of SCAPV notably impacts crop quality and yield.

## Discussion

### Spacial efficiency and environment effectiveness under SCAPV

Improving land space efficiency has been an important indicator in many previous studies on APV,^30^^,^^31^ while the core innovation of SCAPV is to use MPF to create a "photovoltaic umbrella" on plants to improve space efficiency. By coupling photosynthesis and PV through CPV, we efficiently use broad-spectrum sunlight irradiation, especially for light unutilized by plants. The SAPV module expanded solar energy to phytochemical energy and PV energy.

We selected agricultural planting data of species listed in Table 3 and calculated the land equivalent ratio (LER) of ginger, peanuts, sweet potato, bok choy, and lettuce as 1.09, 1.07, 1.17, 1.11, and 1.48, respectively. The highest power generation efficiency of the system in Figure 7C is 11.6%. The highest PCE test result reported for C-Si PV is currently 26.7%;^32^ the calculated LER for the power component of the system was obtained by the ratio of PCE at 0.43 for the same land area and the same PV installation density. Therefore, the total LERs for growing ginger, peanuts, sweet potato, bok choy, and lettuce in the SCAPV system are 1.52, 1.50, 1.60, 1.54, and 1.91, respectively.

(Equation 4.1) $${LER}_{Average}=\left({LER}_{Ginger}+{LER}_{Peanuts}+{LER}_{Sweet\;potato}+{LER}_{Bok\;choy}+{LER}_{Lettuce}\right)\times \frac{1}{5}=1.61$$

The LER of SCAPV with typical crops under SCAPV was calculated as 1.61. The preliminary results of the SCAPV suggested that the LER value, ranging between 1.50 and 1.91, was noticeably higher than 1. This finding implies that an additional 50%–91% land area is required to generate similar amounts of energy and biomass observed in the agricultural system.^26^ Our experimental results, with an LER value of 1.61, further demonstrated the effectiveness of the PV agriculture system in increasing total productivity of land by adopting under-panel planting methods. This finding is consistent with those reported by Valle et al.^33^

Under SCAPV conditions, crops utilized 57% of the solar spectrum but produced higher yields than in open fields, leading to a noticeable improvement in light using efficiency. The increased efficiency is believed to be due to the matching of the transmission spectrum with the absorption spectrum of plant photosynthetic pigments, reducing light inhibition and increasing net photosynthesis accumulation.^34^ Further in-depth research is necessary to understand the mechanism behind the increase.

Soil moisture evaporation was measured through the use of soil evaporation containers.^35^^,^^36^^,^^37^ Although soil evaporation containers have some limitations, such as open containers being susceptible to environmental factors like lateral radiation, which introduce some uncertainty, they have the advantage of using standardized air-dried soils to ensure consistency in initial conditions such as soil composition and moisture. Experiments with soil evaporation containers may therefore provide some valuable insights for reference purposes.

Experimental testing of soil evaporation and micro-environmental monitoring shows that SCAPV can create a better growing environment for plants. The experimental crops grown in the SCAPV environment utilized only a small portion of the solar spectrum but achieved comparable or even better results than those grown in outdoor environments, particularly for root and tuber crops. These findings highlight the effective and efficient coexistence of planting and electricity generation in a shared space. The results suggest that SCAPV systems may revolutionize the way we approach energy production and sustainable agriculture. These systems can generate electricity and create an optimal growing environment for crops via selective spectral filtering. This has significant implications for water conservation, as the SCAPV environment requires less water for crop growth than traditional agriculture. Furthermore, the study highlights the need to research the interdependence of renewable energy and agriculture as we strive to build a more sustainable future. Overall, the study offers valuable insights into the potential of SCAPV systems for enhancing both energy security and food security.

### Selection of crops suitable for SCAPV planting

The cultivation of five crops—ginger, peanut, sweet potato, bok choy, and lettuce—demonstrated an increase in yield under SCAPV compared to open-air cultivation. Notably, unlike other staple food crops, the yield of sweet potato increased by up to 17% under SCAPV compared to open-air cultivation. The adaptability of root and tuber crops, such as ginger, potatoes, and sweet potatoes, makes them well-suited for cultivation under PV, particularly under SCAPV, where the enhanced microclimate induces improved temperature and humidity conditions, mainly caused by the reflection of the infrared radiation that generates heat. Specifically, the growing season for these crops—spring, summer, and autumn—coincides with the period when soil temperatures remain lower and moisture levels higher under SCAPV, creating an ideal environment for harvesting tubers. Moreover, shade-tolerant vegetables, such as bok choy and lettuce, showed an increase in yields under SCAPV, with a notable 10.6% increase for bok choy and as much as 47.9% for lettuce. This finding suggests that SCAPV provides an adequate solution to summer vegetable supply problems in low tropics.

Additionally, we initially selected root crops or shade-tolerant crops that we believed were relatively well-suited to grow under SCAPV conditions and conducted preliminary planting experiments. The preliminary results were positive and provided valuable reference for the subsequent work in terms of crop selection. However, we realize that in terms of agricultural yields and economic viability, the scale and data volume are still far from sufficient compared to traditional field agriculture. Therefore, in the next step, we plan to continuously conduct in-depth experimental verification by selecting a greater variety of crops under the established SCAPV conditions.

### Economic feasibility of SCAPV mass production

Economic efficiency is a crucial factor in determining the viability of PV technology. Conventional SAPV technology prepared optical filters by depositing layers using physical vapor deposition (PVD). However, this method suffers from a slow deposition rate of approximately 0.2–0.5 nm/s, which restricts its applicability in agricultural facilities due to its high production cost.

The present study proposes using the stacking and coextrusion method for developing MPFs. The method yields films that are at the meter level in width and achieve a casting speed of approximately 3,000–5,000 nm/s, a rate which is 15,000 times faster than PVD. Moreover, at one time, the stacking and coextrusion method enables the formation of 128, 256, or more layers of MPF. It is estimated that the cost of mass producing MPF would be no greater than 10 RMB/m^2^. The multilayer coextrusion process offers an advantage in terms of lower production costs for larger areas, making it an important technology for managing agricultural light.

**Figure 10:**
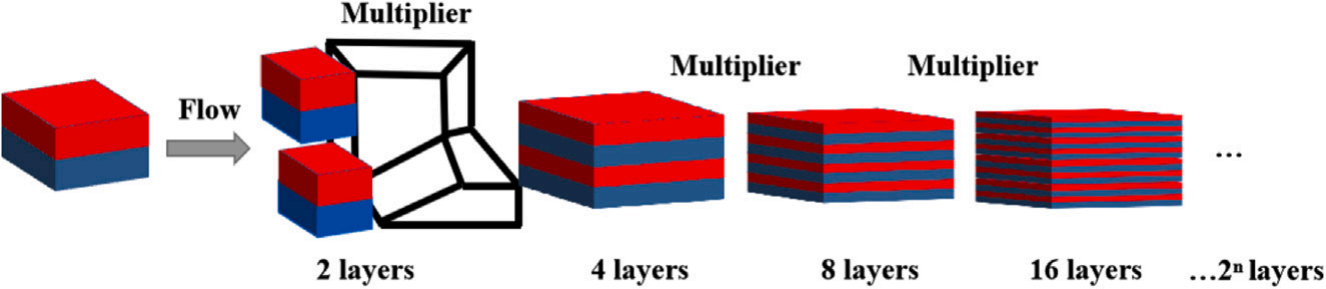
MPF Technical Processes

The MPF consisting of alternate stacking layers of polycarbonate (PC)/polymethyl methacrylate (PMMA) is produced through coextrusion, involving cutting, stacking, spreading, and beginning with two (AB) or 3 layers (ABA) of polymers A and B. The process includes forcing the polymer flows to pass through a series of layer-multiplying elements, resulting in films comprising hundreds of alternate A and B layers, with each layer having a nanoscale thickness is shown in Figure 10. These films entail hundreds of alternating A and B layers, each having a nanoscale thickness, while the total thickness of the material is at the micron scale.

Currently, our team has established a preliminary film production line at 1/20th of the prevailing market cost, based on material expenses.

In recent years, there has been a substantial boost in global installed PV capacity, attributed to the significant reduction in the price of PV modules. Major manufacturers have achieved this reduction by optimizing production equipment and technology routes, leading to a 90% decrease in price. Economically, the SCAPV system introduced in this paper’s customized experimental phase currently lacks a high return on investment (ROI), due, in part, to the procurement of MPF making up more than 50% of the total cost. Nevertheless, the laboratory’s four-pronged plan—mass production of an autonomous film processing process, optimization of mechanical structures, development of lightweight modules, and the realization of scale effect—points toward heightened economic viability. We estimate that when installed capacity reaches 1 GWp, the unit installed cost will drop to 18.8% of the current cost, making SCAPV comparable to ordinary PV in economic terms.

To cope with the problems of mechanized agricultural operations, the longitudinal distance of our SCAPV system can be increased to 7.5 or 9 m by adding one or two concentrator modules, and the height of the prime beam can be raised to 2.8 m to further increase the operating space. This mechanical structure arrangement makes the mechanization of agricultural operations more compatible. In addition, it is worth mentioning that our SCAPV system has an advantage in that the automatic and manual modes can be switched. During sowing and harvesting seasons, the two-axis tracking system is operated manually to allow the concentrator module to be set to a position parallel to the ground, which solves the problem of mechanized operation due to insufficient height at the lowest edge of the module. The SCAPV system was switched to auto-tracking mode during most of the crop growth period.

In addition, cleaning of the reflector is a common and needed problem that can be solved by regular cleaning, anti-dust coating, automatic cleaning system, and regular maintenance. Although this increases the cost of operation and maintenance, it is believed that more effective solutions will emerge with subsequent industrialization studies.

In the future, SCAPV’s affordable economic advantages will also extend to other APV systems, such as extra-high APVs, reaching above 2.8 m above ground, and mosaic-type 50% light-transmitting PV module systems, as presented in Table 4.

**Table 4:** Cost composition of APVs of different types and sizes

Category (RMB/Wp)	Conventional PV	Mosaic APV	SCAPV (1GWp)	Cost reduction factors
PV Brackets	0.36	0.72	0.82	Production at scale; lightweight design; tracking technology maturity increase
PV model	1.9	2.6	0.6	From customization to mass production; increased efficiency of components; market conditions
MPF	0	0	0.11	Film mass production; with glass-free design
Cables	0.32	0.32	0.35	–
Inverter	0.25	0.25	0.25	–
Parallel cage	0.17	0.17	0.17	–
Auxiliary materials	0.15	0.2	0.15	–
Installation	0.3	0.45	0.4	Modular design; Pre assembly
Management	0.27	0.28	0.28	–
Cost	3.72	5.89	3.13	–

SCAPV has the potential to drastically multiply the income per unit area of land by guaranteeing revenue streams from both agricultural products and electricity. For instance, statistics from established experimental sites show that a 1-hectare farm grows up to 32 tons of ginger and generates over 100 MWh of additional electricity, as opposed to 29 tons of ginger in an average farm.

### Potential for SCAPV to improve PCE

#### More accurate MPF for transmission spectra

The main factor contributing to efficiency losses in SCAPV is the MPF. We have demonstrated in the section “Economic feasibility of SCAPV mass production” that it is theoretically possible to obtain films with arbitrary transmission bands. The current article employs an MPF that transmits approximately 57% of the irradiance, but the transmission peak between 710 nm and 852 nm results in a loss of 23.53% of the power of the PV cell. Improving the spectral characteristics of the MPF, by optimizing the bandwidth of red and blue transmission peaks that overlap with the absorption spectra of the plant’s photosynthetic pigments to 60 nm, could increase the efficiency of SCAPV by approximately 30%.

#### Multi-junction cells matched with reflection spectrum

The use of multi-junction cells has been investigated to address the mismatch between the wider solar spectral range and the limited absorption edge limits of a single semiconductor, especially for CPV. However, we found (in part “PV cell selection and efficiency”) that the excellent performance of triple-junction cells in the full solar spectrum did not transfer to the SCAPV after spectral reflection and concentration. We attributed this to the fact that, after spectrum separation by MPF, the light intensity reflected to each junction cell of the triple-junction solar cell differed, resulting in mismatched series current output.

In future upgrades, we plan to either search for multi-junction cells that match the reflection spectrum of the MPF or modify the epitaxial growth structure of the multi-junction based on the reflection spectrum. Additionally, stacked cells made of materials such as III-V semiconductors and chalcogenides are expected to be used for SCAPV systems. Double-junction stacked combinations of III-V top cells and C-Si bottom cells have already achieved conversion efficiencies of 32.8% in the laboratory. Although the cost of these cell technologies is an order of magnitude higher than that of C-Si cells, the advantage of CPV reduces cell usage by an order of magnitude. Spectral separation of SCAPV has the potential to improve performance, provide agroecological benefits, and facilitate the large-scale application of related technologies in the future.

#### Improve the accuracy of DATS

Improving the accuracy of DATS is critical to enhancing efficiency, particularly for the large-scale promotion of APV stations. Our goals include mechanical mechanism stability, controllable costs, and high accuracy. However, the current mechanical structure of the system is suboptimal, as it only uses astronomical tracking algorithms. As a result, we plan to increase the adjustment step of the gyratory gear motor, simplify the tracking mechanism parts, and add optical and astronomical algorithms in the next upgrade to improve the system efficiency and achieve our goals.

### Conclusion

The integration of PVs and photosynthesis through spectral separation on the same land offers a promising solution to the food crisis and energy transition problems faced by countries such as China with unbalanced population and energy distribution. Although SAPV research is still in its prototype and conceptual stage, a sustained breakthrough is required to improve PCE, plant growth, and microclimate on a large scale.

This study reports, for the first time, a large-scale and cost-efficient SCAPV, with maximized PCE of the system increased to 11.6%. Modular design of the concentrator module, optimization of cell components and concentrating curve, and optimization of the DATS were used to achieve this efficiency, which is the highest PCE reported in the literature regarding spectral separation technology. Combining CPV and multi-junction solar cells through MPF has the potential of higher comprehensive efficiency. A 400 m^2^ power plant with an installed capacity of over 10 kWp was built in Anhui, China, with an average annual power production of over 107 MWh/ha. Field planting experiments showed that five crops (ginger, peanut, sweet potato, bok choy, and lettuce) had an average yield increase of 18.4% under SCAPV compared to open air, along with an LER of 1.61, indicating that the total land productivity could be effectively increased with this planting scheme. The study findings also discussed the crop types suitable for planting under SCAPV, and the results revealed that root and shade-tolerant crops yielded better. The microclimate at the bottom of the SCAPV system, especially the soil moisture retention capacity in the daytime, was better than in the open air. This demonstrates the agricultural friendliness of SCAPV technology at scale and avoids achieving global energy transition goals at the expense of food production or biodiversity. Besides, this study’s construction is the first large-scale outdoor SAPV experimental facility, and the positive results of crop and microclimate studies have implications for other spectral separation solutions. Compared with conventional PV power stations, it is estimated that the cost of spectral separation PV agricultural systems is expected to decrease by 18.8%.

Our results show that spectral separation PV technology has significant potential in combining photosynthesis and PVs.

### Limitations of the study

Further research is needed for more quantitative and refined agricultural experiments. The article does not delve into the physiological mechanism of the impact of red and blue light transmission on plants, and it needs to be explored.

## STAR★Methods

### Key resources table

**Table:** 

REAGENT or RESOURCE	SOURCE	IDENTIFIER
**Software and algorithms**		

GraphPad Prism	10.0.2 (171)	https://www.graphpad.com
Microsoft PowerPoint	16.76(23081101)	https://www.microsoft.com
Solidworks	2017 SP3.0	www.solidworks.com.cn/

**Other**		

Solar Module Analyzer	PROVA200	https://www.tes.com.tw
Solar Power Meter	TES-1333	https://www.tes.com.tw
Meteorological Stations	RS-QXZ	http://www.rkonfly.cn
Ocean Insight	SR2	https://www.oceaninsight.com
LI-COR	LI-190R	https://www.ecotek.com.cn

### Resource availability

#### Lead contact

Further information and requests for resources should be directed to and will be fulfilled by the Lead Contact, zhangxy@ustc.edu.cn.

#### Materials availability

Any additional information required to reanalyze the data reported in this work paper is available from the lead contact upon request.

### Method details

#### Power conversion efficiency

The concentrated PV module's power was tested at various times (TES PROVA200 solar cell analyzer, China). The formula for calculating PCE is as follows:^38^

$$PCE=\frac{I_{sc}\cdot V_{oc}\cdot FF}{P_{sun}}=\frac{\max\left(V\cdot I\right)}{\int_{0}^{\infty }G\left(\lambda \right)d\lambda }$$

Where Isc, VOC and FF are the short circuit current, the open circuit voltage and the fill factor respectively. G(λ) is the AM1.5 spectrum and Psun is the total energy power integrated over the entire solar spectrum (AM1.5 spectrum). Real-time irradiance was measured by a solar power meter (TES-1333, China). Normalizing the solar irradiance to 1000 W/m^2^ (according to the IEC 61215-1 standard), one can determine the PV efficiency of the SCAPV system. The PCE of GaAs was tested similarly to the method described above.

#### The spatial efficiency evaluation

Combining PV modules and crops on a single land area increases its productivity. The land equivalent ratio (LER) is a term used to evaluate this increased land productivity. LER is suitable to evaluate SCAPV performance under mixed cultivated patterns.^33^^,^^39^ It is defined as:

$$\text{LER}=\frac{{\text{Crop}\;\text{Yield}}_{\text{SCAPV}}}{{\text{Crop}\;\text{Yield}}_{\text{natural}\;\text{state}}}+\frac{{\text{Electricity}}_{\text{SCAPV}}}{{\text{Electricity}}_{\text{PV}\;\text{Station}}}$$

In the above equation, the ratio of electricity of SCAPV and PV Station is equivalent to the ratio of power conversion efficiency of them for the same land area and the same PV installation density.

#### Water evaporation and microclimate

The experimental design procedures and data analysis for soil water evaporation under SCAPV are described in Supplementary Material S3.

Air-dried loamy soil was procured from Huabiao (Chengdu, China). The soil was dried for 78 hours and then carefully mixed to distribute uniform moisture content. The air-dried soil was then compacted to a depth of 25 cm inside the evaporation containers. The soil-filled evaporation containers were immersed into water with a depth of 10 cm for 48 hours. Subsequently, the evaporation containers were removed from the water tank to discharge gravity water through the bottom holes for 24 hours.^40^ Thereafter, the evaporation containers were weighed and placed in (a) open-air and (b) SCAPV, as in Figure S2. Three replicates were used in each experiment.

Environmental parameters including air temperature, solar radiation, relative humidity, and wind speed are measured by multi factor meteorological stations (RS-QXZ, Renke, China). The absolute spectra from 300 nm to 900 nm and PPFD were measured by micro-spectrometer (Ocean Insight SR2, USA) and Li-190R (LI-COR, USA), respectively. The PPFD of P1, P2, P3 under SCAPV is measured and averaged by li-190R at multiple points on clear weather.
