# Supplementary material for: Large-scale and cost-efficient agrivoltaics system by spectral separation
Source: iScience. 2023 Oct 5;26(11):108129. doi: 10.1016/j.isci.2023.108129 (PMC10590749; doi:10.1016/j.isci.2023.108129)
Supplement: Document S1. Figures S1 and S2 and Table S1 [file mmc1.pdf]

iScience, Volume *26*

Supplemental information

Large-scale and cost-efficient agrivoltaics

system by spectral separation

Fangxin Zhang, Ming Li, Wei Zhang, Wenjun Liu, Altyeb Ali Abaker Omer, Zhisen Zhang, Jianan Zheng, Wen Liu, and Xinyu Zhang

Supplementary Figures

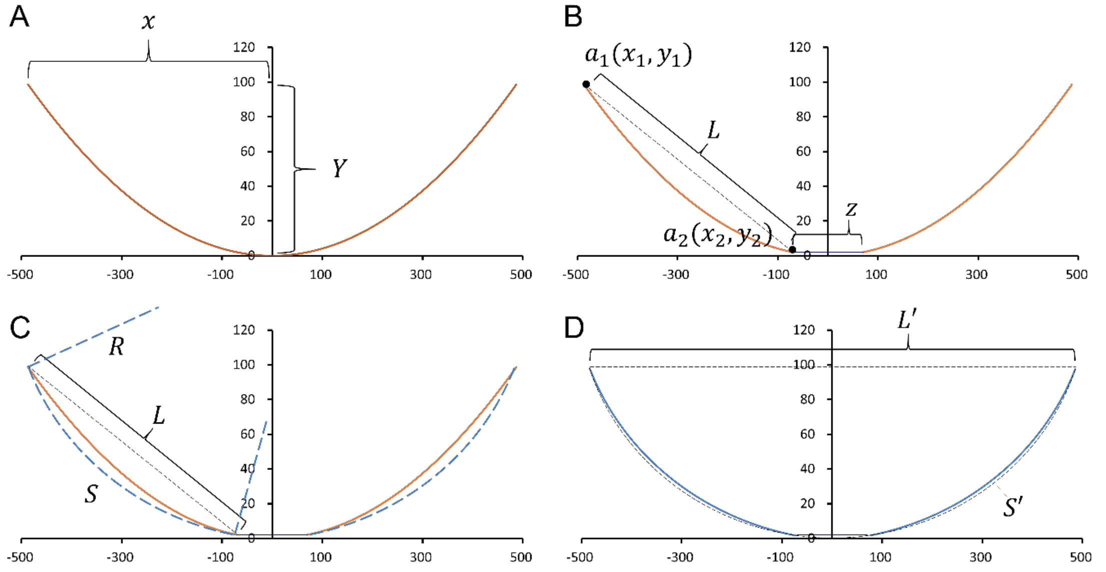

Figure S1. Steps for calculating the emitter mirror curve for a concentrator module. (A) Ideal parabolic curve parameters; (B) Parabolic curve for the middle opening after taking into account PV shading; (C) Parabolic curve substituted with circular arc; (D) Final production reflector curve, related to Figure 2.

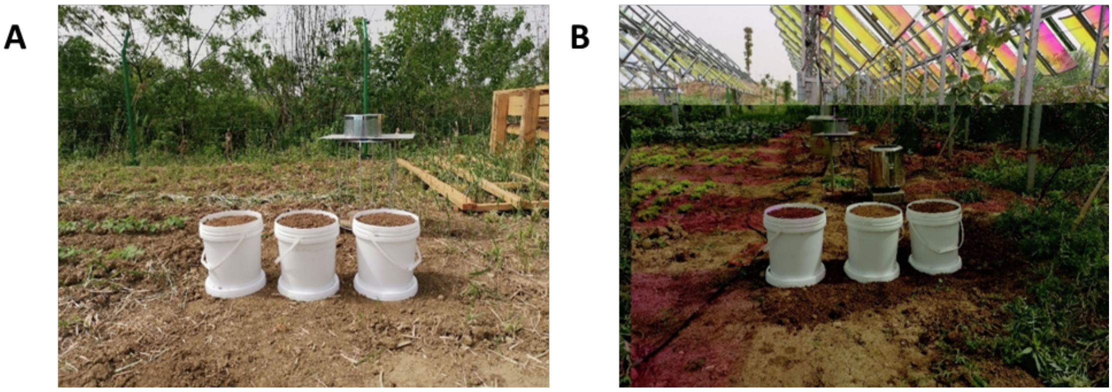

Figure S2. The design of two experimental treatments: the evaporation containers and pans evaporation placed (A) open-air and (B) SCAPV, related to Figure 9.

Supplementary Tables

Table S1. Information Table of Crop Planting Experiment

Crop	Planting time	Planting area (m^2)	Row and plant spacing	Fertilizer
**Ginger**	2019/4 - 2019/10	100	0.5*0.5	Compound fertilizer 20 kg/mu
**Peanuts**	2020/4 - 2020/9	15	0.35*0.25	Compound fertilizer 20 kg/mu
**Sweet potato**	2020/5 - 2020/10	18	0.9*0.25	Compound fertilizer 4.5 kg/mu
**Bok choy**	2021/3 - 2021/5	8	0.2*0.2	Compound fertilizer 50 kg/mu
**Lettuce**	2021/3-2021/5	8	0.2*0.2	Compound fertilizer 50 kg/mu

Table S1. Information Table of Crop Planting Experiment, related to Table 3
